# Synchrotron-based FTIR microspectroscopy of protein aggregation and lipids peroxidation changes in human cataractous lens epithelial cells

**DOI:** 10.1038/s41598-020-72413-9

**Published:** 2020-09-23

**Authors:** Martin Kreuzer, Tanja Dučić, Marko Hawlina, Sofija Andjelic

**Affiliations:** 1 CELLS–ALBA, Carrer de la Llum 2-26, Cerdanyola del Valles, 08290 Barcelona, Spain; 2 Eye Hospital, University Medical Centre, Grablovičeva 46, 1000 Ljubljana, Slovenia

**Keywords:** Lens diseases, Infrared spectroscopy

## Abstract

Cataract is the leading cause of blindness worldwide but the mechanisms involved in the process of cataractogenesis are not yet fully understood. Two most prevalent types of age-related cataracts are nuclear (N) and cortical (C) cataracts. A common environmental factor in most age-related cataracts is believed to be oxidative stress. The lens epithelium, the first physical and biological barrier in the lens, is build from lens epithelial cells (LECs). LECs are important for the maintenance of lens transparency as they control energy production, antioxidative mechanisms and biochemical transport for the whole lens. The purpose of this study is to characterize compounds in LECs originated from N and C cataracts, by using the synchrotron radiation-based Fourier Transform Infrared (SR-FTIR) microspectroscopy, in order to understand the functional importance of their different bio-macromolecules in cataractogenesis. We used the SR-FTIR microspectroscopy setup installed on the beamline MIRAS at the Spanish synchrotron light source ALBA, where measurements were set to achieve single cell resolution, with high spectral stability and high photon flux. The results showed that protein aggregation in form of fibrils was notably pronounced in LECs of N cataracts, while oxidative stress and the lipids peroxidation were more pronounced in LECs of C cataracts.

## Introduction

Cataract that can be defined as any opacity of the crystalline lens is the leading cause of blindness worldwide accounting for 48% of all causes of blindness. According to the World Health Organization, it is estimated that about 20 million people today have bilateral visual impairment due to cataract, this number is expected to reach 50 million by 2050^1^. However, the mechanisms involved in cataractogenesis, the process of cataract formation, are not yet fully understood. Based on the location of the opacity, cataract can be divided into cortical (C), nuclear (N) and posterior subcapsular cataract. Lens is build from two type of cells, lens epithelial cells (LECs) and fiber cells, the second making up the substance of the lens, both its cortex and nucleus.

The single-layered lens epithelium underlies the anterior capsule on the anterior lens surface. Being located between the aqueous humour and the lens fibre cells, it is the first physical and biological barrier in the lens and is important for protecting the lens interior. It is metabolically the most active part of the lens, which maintains the lens physiological health. The majority of the homeostatic functions of the lens are regulated by LECs. LECs control energy production, antioxidative defence mechanisms and biochemical transport for the whole lens, and their integrity is important for keeping of lens transparent^2^.

Cataract formation can be induced by different factors: oxidative stress, UV or other toxic agents^3^. Oxidative stress is an usual environmental factor in most age-related cataracts^4,5^. Cataractogenesis could occure when the rate of reactive oxygen species (ROS) production is superior to the rate of their removal^6^*.* Environmental stress can cause substantial increase of ROS levels. Entry of light into the eye contributes significantly to the cataractogenesis, mostly through photochemical generation of ROS and subsequent oxidative stress to the tissue^7^*.* ROS can cause DNA, RNA, lipids and proteins damage. One opinion is that the free radicals high reactivity induce oxidative damage of fiber-cell membranes and proteins of the lens, beginning the progression of age-related cataract^8,9^. The lipids peroxidation, the oxidative degradation of lipids, which can be initiated by increase of oxygen free radicals in the eye fluids and tissues and reduced lens antioxidant defences, may also lead to cataractogenesis^10^. Besides, lens lipid composition alters importantly in cataract^11^. These changes could lead to crystalline structure of the lens, disturbed with age, the oxidative damage accumulates in the lens, which decrease turnover of lipids or proteins in the lens^12,13^. Nucleic acids are also prone to these changes, as oxidative stress and changed antioxidative defense capacity also modify the rate of telomere shortening^14^.

As LECs have exterior position in the lens, they are the first lens cells exposed to diverse environmental factors that contribute to the cataractogenesis. LECs high metabolic activity makes them subject to oxidative damage, and in UV-induced lens damage one of the early events is the lens epithelial lipid oxidation^15^. Cortical cataracts are related to mutations and lens epithelial changes, especially after ultraviolet (UV) and infrared (IR) exposure^16^. UV radiation induced cataract begins with the lens epithelial damage that includes membrane permeability change, with a consequence of loosing the ions homeostasis within the lens^15^. UVB-induced cataract starts with LECs damages triggering apoptosis^17,18^. From the other side, it is believed that human LECs apoptosis is an initiating element in cataract development^3,19^. Pathogenesis of UV-induced cataract and molecular pathways involved in apoptosis were reviewed by Kamari et al.^20^. Although LECs are equipped with machinery to combat with cataractogenic insults, any alteration in the lens epithelium may proceed further in the remaining part of the lens and may lead to cataract^21^.

As all bio-macromolecules could be involved in cataracts, here we employed the SR-FTIR microspectroscopy to study the chemical composition information of a N and C cataract samples and to provide their molecular fingerprint. FTIR is a vibrational spectroscopic technique that is a powerful tool for the cell components analysis, such as nucleic acids^22^ proteins^23^ and membranes^24^. The analysis of spectral data provides qualitative and quantitative information of a cell component on the basis of peak's shifts, bandwidths and band intensities.

Previously, FTIR microspectroscopy was used to study the whole lens^25–29^. However, FTIR spectroscopy and microscopy were not used to study human LC LECs until now, up to our knowledge. Lens epithelium was studied by Raman spectroscopy after exposure to a low-dose-range of ionizing radiation in order to identify early predictors of lens degeneration resulting in cataractogenesis^30^, otherwise the Raman microspectroscopy was also mostly used for studying the whole lens.

In this study, biochemical differences between lens epithelia from N and C cataracts were studied in-situ by means of SR-FTIR spectroscopy and microscopy. Spectral distinctions were followed by principal component analysis. We evaluated the proteins conformal changes, lipids oxidative stress and nucleic acids changes in LECs of N and C cataract types. Besides spectroscopical analysis, several areas of two patients lens epithelia were investigated by FTIR imaging, in order to locate possible changes within individual anterior lens epithelium.

## Material and methods

### Samples

Sample preparation follows the procedure described in^31^ and is summarized below. Experiments were performed on the anterior LC preparations consisting of the monolayer of LECs attached to the basement membrane, i.e. the capsule matrix. The LCs were obtained routinely during cataract surgery performed at the Eye Hospital, University Medical Centre in Ljubljana, Slovenia. We examined 12 patients’ LCs. The central LECs were studied from the approximately 5–5.5 mm circles of the central anterior LCs that were carefully removed by continuous curvilinear capsulorhexis^31^. The material originated from 12 different cataract patients, 5 male and 7 female, whose age was between 31 and 88 with the average being 67 years. After the surgery, each LC was stored in high glucose medium (DMEM; Sigma, No. 5671, St. Louis, MO, USA) supplemented with 10% FBS and 1% antibiotics (penicillin–streptomycin; Sigma, No. 4333), and transported to the laboratory at Eye Hospital, University Medical Centre in Ljubljana, Slovenia.

The samples obtained during cataract surgery of patients with different cataract types and degrees were first rinsed in 5 ml NaCl for 10 min and then placed by gently stretching and plating adherently on circular 13 × 0.5 mm CaF_2_ slides (Crystan Ltd., UK) by using micro-dissecting tweezers (WPI byDumont, Med.Biologie, Germany). The samples were dried under sterile conditions in the laminar flow at room temperature and stored over silica gel prior the measurements at the ALBA synchrotron.

### Synchrotron based FTIR

To assess the organic compounds profiles, measurements at the infrared microspectroscopy beamline MIRAS at the ALBA synchrotron light source (Barcelona, Spain) have been performed^32^. Although conventional FTIR spectroscopy is a valuable tool for examining larger cell populations in the tissues, the limited brightness of standard infrared light sources generally precludes high spatial (single-cell) resolution measurements^33^.

All SR-FTIR microspectroscopic absorption spectra were collected in transmission mode using the infrared microscope Hyperion 3000 coupled to a FTIR Vertex 70 spectrometer (Bruker, Germany) using a liquid nitrogen cooled mercury cadmium telluride (MCT) detector. Each spectrum was acquired after co-adding 256 scans at 4 cm^−1^ spectral resolution. We used the OPUS 7.5 (Bruker) software package for data collection.

The spectral analysis was focused on the wavenumber regions of phosphates (1,000–1,300 cm^−1^), i.e. nucleic acids, Amide I and II (1,480–1,700 cm^−1^), i.e. proteins, lipids (2,800–3,000 cm^−1^) and the carbonyl group (1,730–1,760 cm^−1^), i.e. ester compounds.

The oxidative stress was estimated by following the lipids peroxidation by using the ratio of lipidic bands: asymmetric vibrations of CH_2_ and CH_3_ (ν_as_ CH_2_/ν_as_ CH_3_) (A_2925_/A_2960_), as well as by ratio of carbonyl groups to asymmetric bands of CH_2_ and CH_3_ (A_1740_/A_2960+2925_).

**Figure 1:**
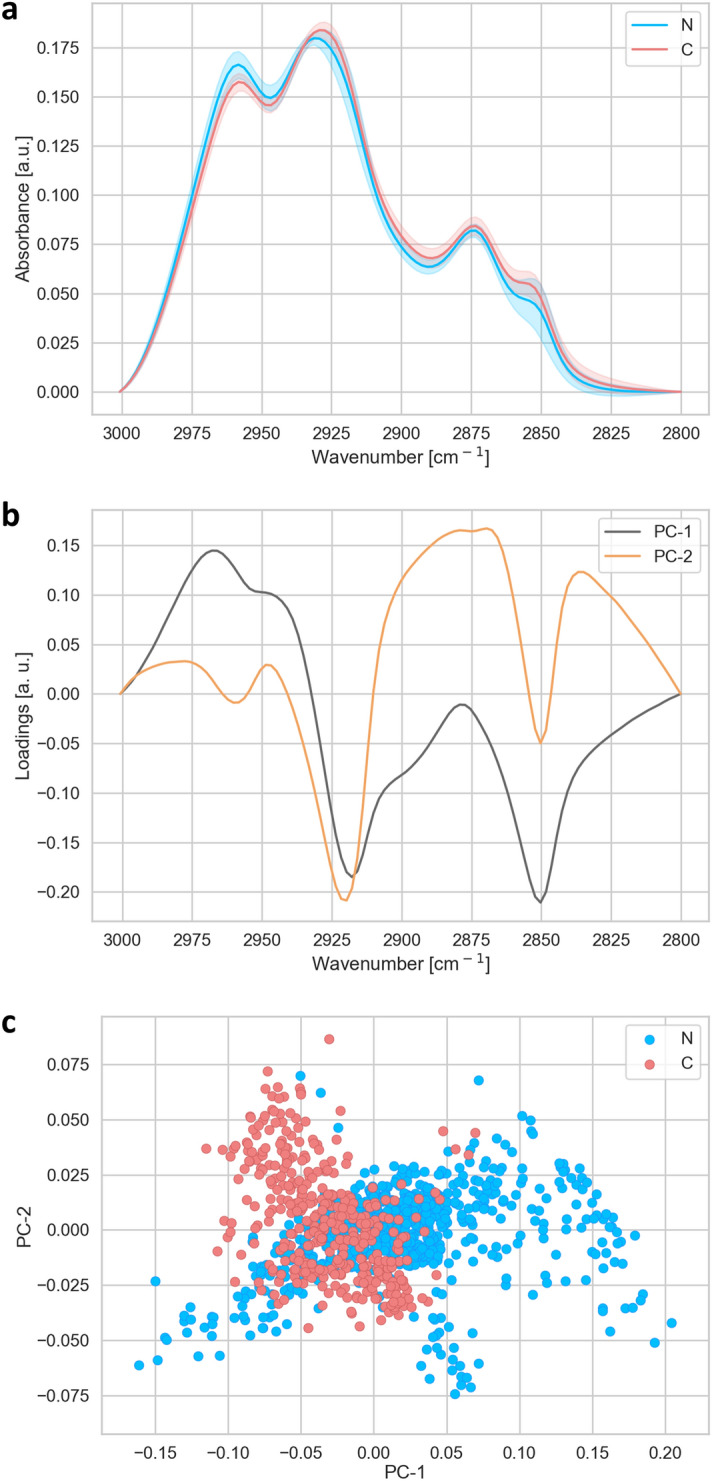
Analysis of the lipid region (2,800–3,000 cm^−1^). (**a**) Average FTIR spectra of N-(blue) and C-(red) types of cataract. (**b**) PCA loadings of the first two components (PC1 black and PC2 orange), representing 79 and 12% of the total variability of the samples, respectively. (**c**) PCA scores plot denotes the variability associated with the first two components.

**Figure 2:**
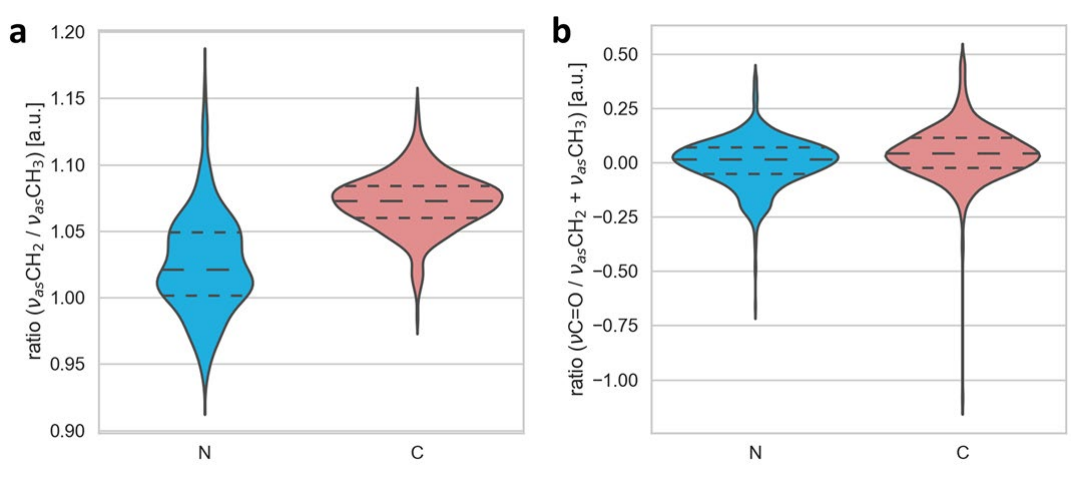
Analysis of oxidative stress markers. Distribution of the ratio between the asymmetric CH_3_ and CH_2_ bands (**a**) and the ratio between the C=O band and the sum of asymmetric CH_3_ and CH_2_ bands (**b**) for N-type (blue) and C-type (red) cataract. Values are presented with the probability density of the data at different values and mean ± SD.

**Figure 3:**
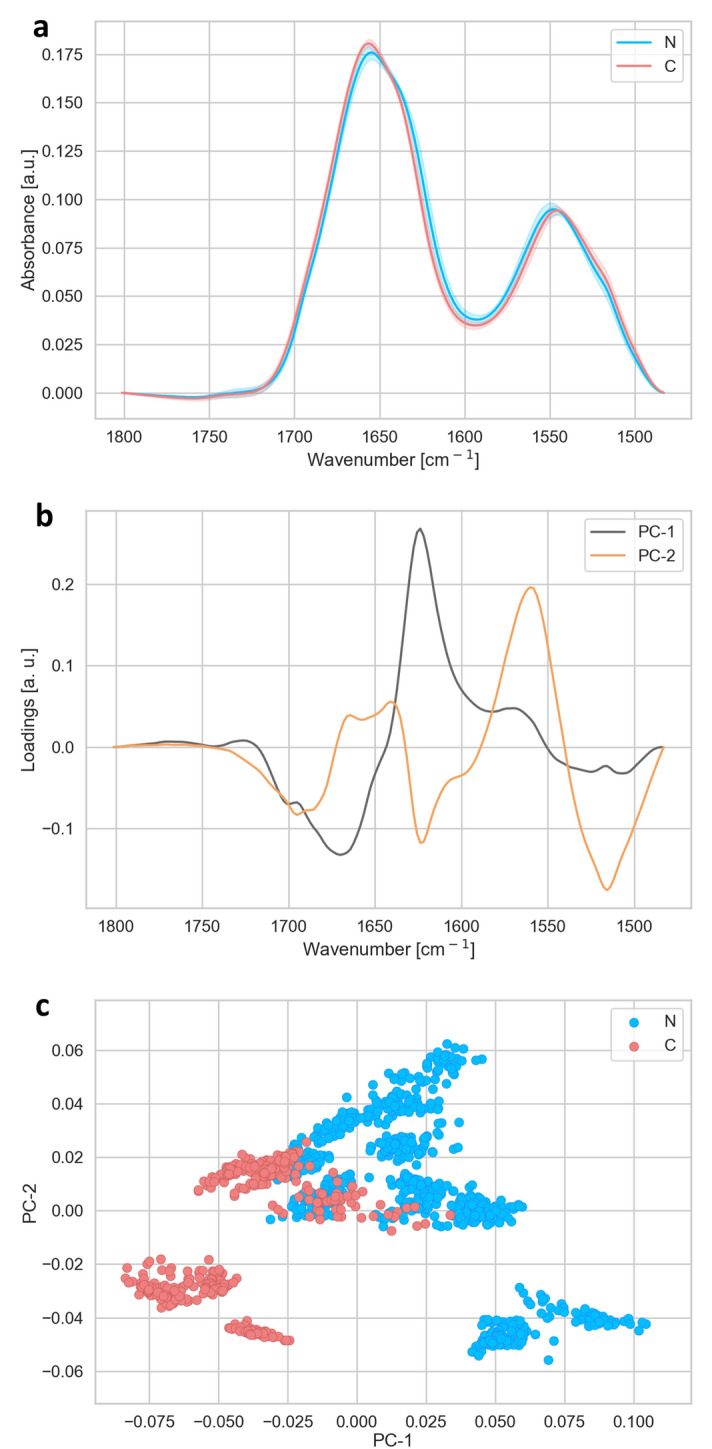
Analysis of the spectral region of Amide I and Amide II and carbonyl region (1,480–1,800 cm^−1^). (**a**) Average FTIR spectra of cells for N-(blue) and C-(red) types of cataract. (**b**) PCA loadings of the first two PCA components (PC1 in black and PC2 in orange), representing 59 and 30% of the samples variability, respectively. (**c**) PCA scores plot denote the variability in the first two components.

**Figure 4:**
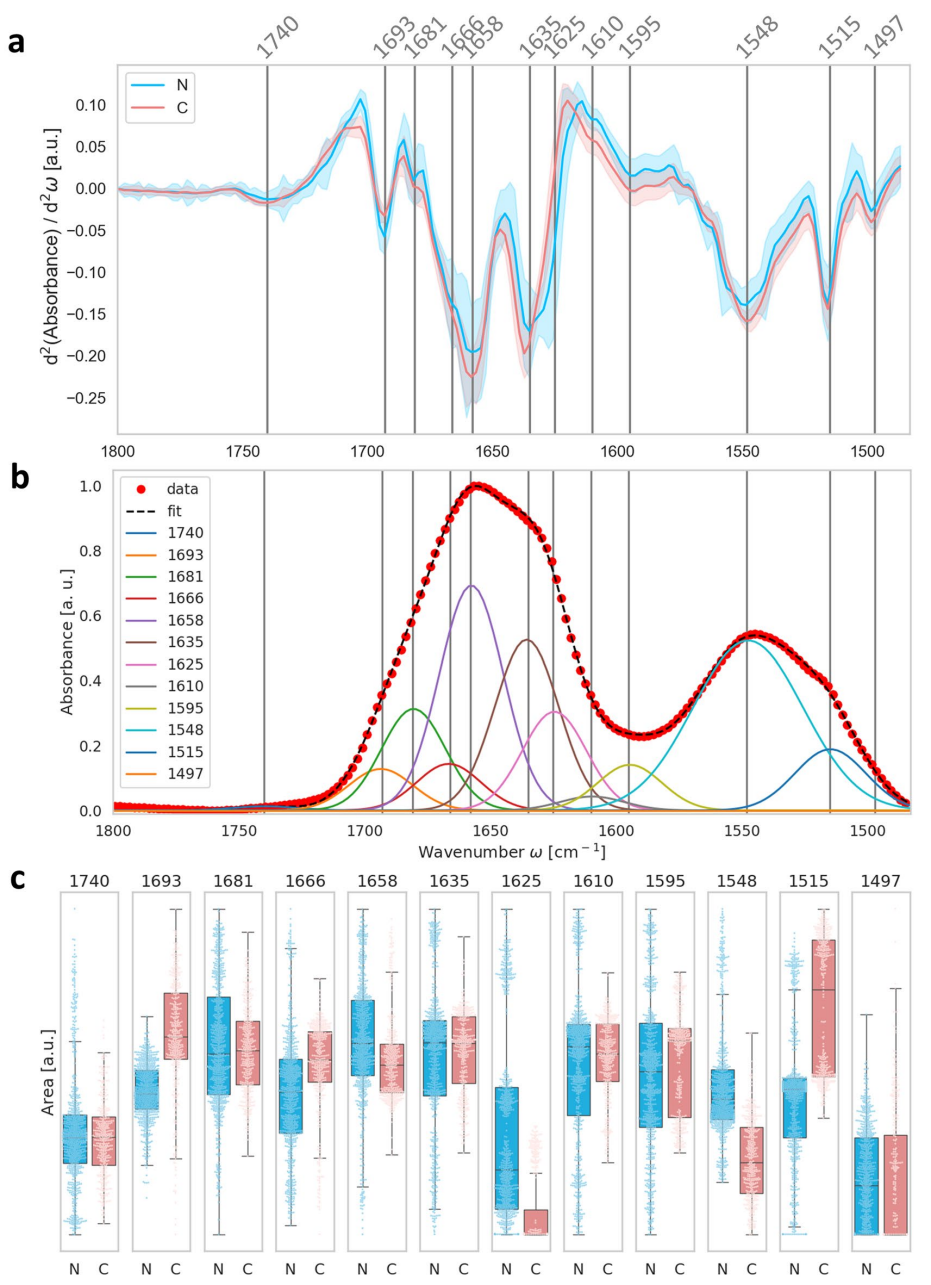
Band deconvolution of the Amide I and II and carbonyl region (1,480–1,800 cm^−1^). (**a**) Averages of second derivatives; (**b**) Deconvoluted spectra of the spectral region with Gaussian curves. The number and Gaussian curves maxima positons have been approximated from the minima positions of the second derivatives. In the fitting routine the band widths were fixed to 30 cm^−1^ in the Amide I region. (**c**) Box plots of the areas below each Gaussian curve for N-type (blue) and C-type (red) cataract, representing the carbonyl group (1,740 cm^−1^), oligomers (1,693 cm^−1^), turns and loops (1,681 and 1,666 cm^−1^), α helix (1,558 cm^−1^), β-sheet (1,635 cm^−1^), cross-β-sheet (1,625 cm^−1^), side chains such as Tyr and Asn (1,610 cm^−1^), side chain as Tyr, Glu, and Asp residues (1,595 cm^−1^), Amide II (1,548 cm^−1^), Tyr (1,515 cm^−1^) and aromatic ring vibration (1,497 cm^−1^). Values are presented with overlapping swarmplots, showing the distribution of the values.

**Figure 5:**
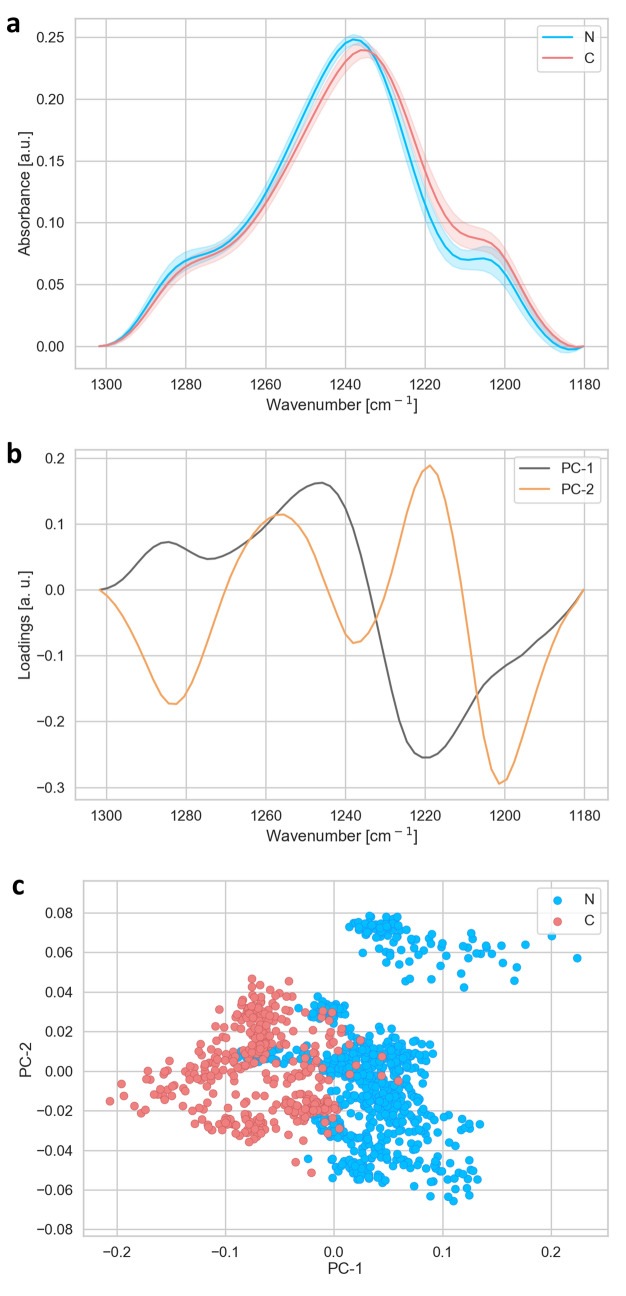
Analysis of the spectral region of phosphates bands of nucleic acids in the wavenumber region (1,180–1,300 cm^−1^). Average spectra (**a**) of N-(blue) and C-(red) types of cataract and corresponding loadings plot (**b**) with PC1 (black) and PC2 (orange), representing 73 and 16% of the total variability of the samples, respectively. The PCA scores (**c**) plot denotes the variability associated with the first two components.

**Figure 6:**
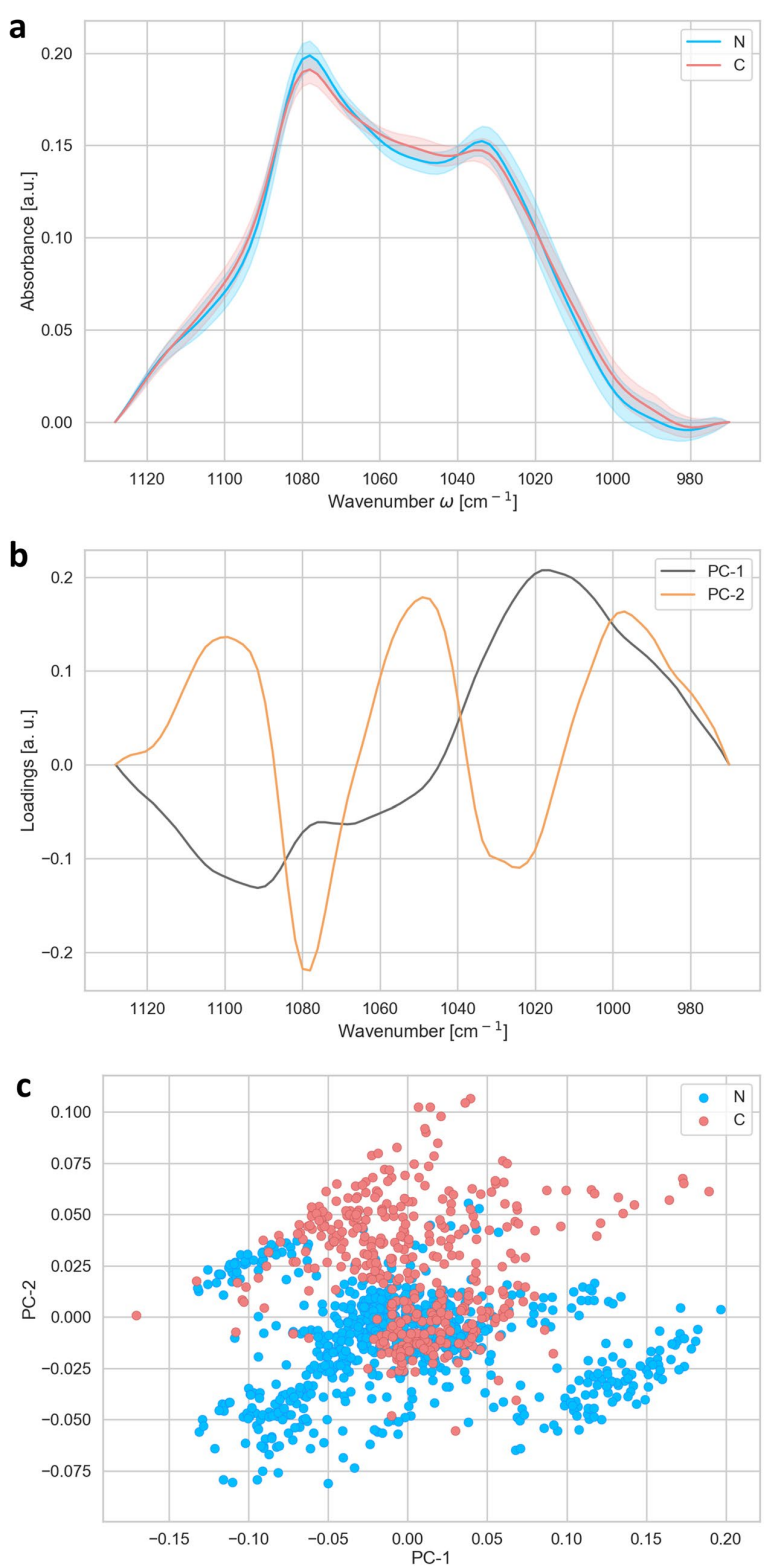
Analysis of the spectral region of phosporilated proteins and C–O stretch of ribose ring (970–1,130 cm^−1^). Average spectra (**a**) of N-(blue) and C-(red) types of cataract and PCA loadings plot (**b**) of the same spectral region with PC1 (black) and PC2 (orange), explaining 68 and 18% of the variability among the sample, respectively. PCA scores plot (**c**) denotes the variability of the samples associated with the first two components.

In order to achieve the single cell data acquisition and analysis (Figs. 1, 2, 3, 4, 5, 6), we acquired spectra of 10 × 10 μm^2^ areas of the tissue, by using the aperture of the microscope. Two different regions of each sample have been measured: one region close to the centre and one region close to the periphery of the each sample. For statistical analysis, in each region a matrix of 7 × 7 spectra has been measured, i.e. 49 in total, resulting in a measured area of 4,900 μm^2^ per region. In total 1,127 spectra were analysed: for N1 245 spectra, N2 98 spectra, N3 196 spectra, N4 196 spectra, C1 294 spectra and C2 98 spectra. N corresponds to the nuclear type of cataract and the C to the cortical type of cataract, with the number corresponding to the degree of cataract development, where 1 is the lowest and 4 is the highest level of cataract development. Total number of spectra analyzed was 735 N and 392 C.

In order to work with more compact i.e. small light path length, we divided spectra in four parts as shown in Figs. 1, 3, 5 and 6. Each part of spectra was separately baseline corrected and subsequently vector normalized. The baseline correction and unit vector normalization in each region of interest were done by using ORANGE software (Bioinformatics Laboratory of the University of Ljubljana^34^, Version 3.20.1, with the spectroscopy package^35^ (Version 0.4.1). The same software was also used to perform PCA analysis on the different wavenumber regions.

Furthermore, the Amide I and II bands (1,480–1,700 cm^−1^) were deconvoluted for each spectrum by using a Python script using numpy, scipy and seaborn libraries. For each spectrum the region was fitted using 12 Gaussian functions. The centre positions of the Gaussians were derived from the minima positions of the second derivative in that wavenumber region and could vary  ± 0.5 cm^−1^ in the fitting routine. The second derivatives were calculated with a window size of 5 and polynomial order of 2. The full width at half maximum was set fixed to 30 cm^−1^ for the Gaussians underlying the Amide I band. The resulting areas underlying the Gaussian bands were calculated for each spectrum and plotted in a boxplots with overlapping swarm plots, showing the deviation of the results. Table 1 shows band assignment for Amide II, Amide I and carbonyl group, according to^36,37^.

**Table 1:** The FTIR bands of Amide I and II assignment according to literature values, as described in the text.

Band assignment	Peak [cm^−1^] This study	Peak [cm^−1^] Literature
**Amide II**		
Tyrosine	1,515	1,515^38^
α-Helices	1,548	1,550^38^
**Amide I**		
Side chains	1,610	1,606^36^
Cross β-sheets	1,625	1,620^36^
Parallel β-sheets	1,635	1,637^36^
Unordered structures	1,645	1,646^36^
α-Helices	1,658	1,654^36^
Loops and turns	1,666	1,668^36^
Anti-parallel β-sheets	1,693	1,697^36^
**Carbonyl group**		
Ester	1,740	1,730–1,740^39^

### FTIR imaging data acquisition and analysis

**Figure 7:**
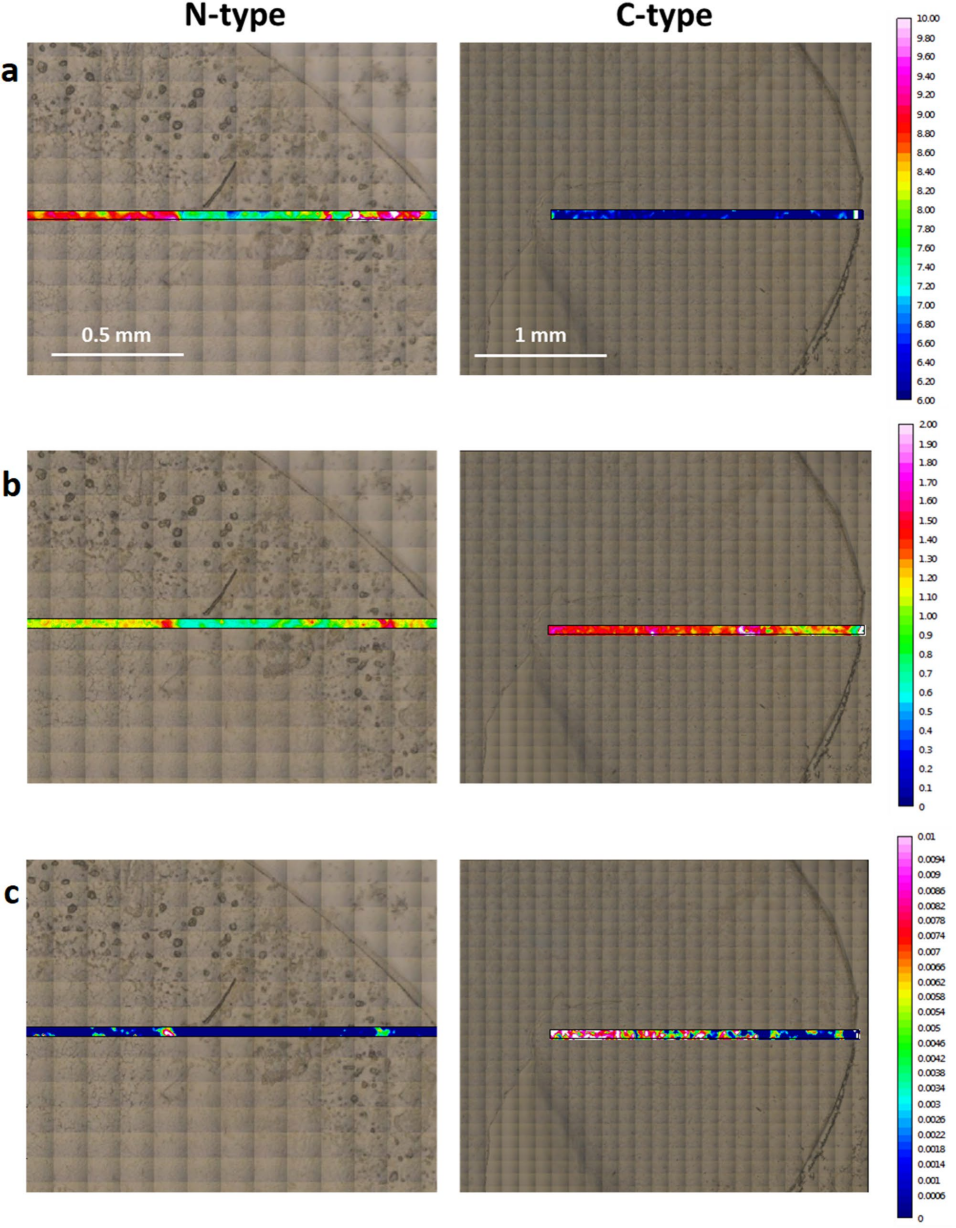
The FTIR images of the regions of interest (in color) are superimposed on the visible light bright field micrographies (field of view). Chemical maps in colors of N-type (stage 4) and C-type (stage 2) samples based on the integrated absorbance between (**a**) 1,630 to 1,620 cm^−1^ represents proteins fibril aggregates, (**b**) the ratio between ν_as_CH_2_ (2,945–2,900 cm^−1^) and ν_as_CH_3_ (2,990–2,945 cm^−1^) and (**c**) the ratio between νC=O (1,760–1,730 cm^−1^) and ν_as_ (CH_2_ + CH_3_) (2,990–2,900 cm^−1^) representing lipid peroxidation ratios.

Matrices with 160 × 800 spectra were measured with an aperture size of 10 × 10 µm^2^ and a stepsize of 10 µm (Fig. 7). The total absorbance cartograms were generated by integrating spectral regions of interests. FTIR imaging was done on 2 patients’ LCs, which were randomly selected: C2-from a 31 years old female patient (right column) and N4-from a 76 years old female patient (left column).

### Ethics statement

The research followed the tenets of the Declaration of Helsinki. The study was approved by the National Medical Ethics Committee of the Republic of Slovenia and all patients signed informed consent before the operation.

## Results

### Single cells analysis

All measured spectra have been divided in the two groups N-type and C-type cataract. Biochemical differences have been reported for different wavenumber regions: lipids, proteins and nucleic acids.

Figure 1a shows FTIR average spectra with standard deviations obtained from the lipid region (2,800–3,000 cm^−1^). The average plot shows the most pronounced absorption bands with maxima at 2,960 cm^−1^ and 2,925 cm^−1^, which correspond to the asymmetric vibration of CH_3_ and CH_2,_ respectively. The bands with maxima at 2,875 cm^−1^ and 2,850 cm^−1^ correspond to the symmetric vibration of CH_3_ and CH_2,_ respectively. A PCA analysis of this lipid region is shown in Fig. 1 with the loadings plots (B) and the PCA score (C). The analysis reveals that the two cataract types show distinct spectral features, as can be seen in the separation of the two groups in the PCA scores plots. Pronounced changes in the lipid region were observed in asymmetric vibration of CH_2_ and symmetric vibration of CH_2_. In addition, the ratio of the asymmetric vibrations CH_2_ and CH_3_ (ν_as_ CH_2_/ν_as_ CH_3_), as a marker of the oxidative stress, i.e. indicators of lipids peroxidation^40–42^, showed an increase in C-type of cataract (Fig. 2a). The second parameter for the oxidative stress, the ratio of the carbonyl group and asymmetric vibration of both CH_2_ and CH_3_ (*ν* C=O/νas CH_2_ + CH_3_) showed small increase in C-type of cataract (Fig. 2b). However, regarding the other lipid peroxidation stress markers as a band at 3,010 cm^−1^ (corresponding to C–H stretching close to C=C) and lipid peroxides band C–O stretching bond at 1,260 cm^−1^^43^, we did not observe the differences.

The most pronounced part of the proteins in Amide I and Amide II regions including the ester groups (1,480–1,800 cm^−1^) are displayed in Fig. 3a. As the protein bands with maxima at 1,655 cm^−1^ (Amide I) and 1,545 cm^−1^ (Amide II) are sensitive to changes in protein secondary conformation, these bands were investigated further by PCA. The first component of the PCA (Fig. 3b) showed the most prominent contribution of the band at 1,625 cm^−1^ in the Amide I region. This peak is associated with intramolecular beta sheet organisation in protein fibrils aggregates. The PCA analysis showed that the N-type of cataract contained more beta sheet organisation than the C-type of cataract. From the minimum position of PC1 in the loadings plot (Fig. 3b) we noticed that the C-type of cataract in general contained more turns and loops regarding their secondary proteins structure (maxima at 1,670 cm^−1^). The PC2 laodings plot (Fig. 3b) pointed differences in Amide II at ~ 1,560 cm^−1^ and ~ 1518 cm^−1^ associated with shift toward beta sheets and Tyrosine amino acid residue^36,37^. Figure 3a,b pointed out that the most of N samples shows the small shoulder peak at ~ 1,515 cm^−1^, which indicated that these cells contained proteins with Tyrosine residues. The analysis shows that there are clear differences in protein secondary contribution between N and C-type of cataract.

In order to analyse the observed changes in the secondary protein structure in the two types of cataract in more detail, we calculated the second derivative of the protein region (Fig. 4a), assigned the minima positions and used them as centre positions of Gaussian curves to perform a band deconvolution of the protein region for each spectrum. Figure 4b shows the deconvolution of a single spectrum as an example. The assignment of the identified bands within the Amide I band (1,600–1,780 cm^−1^) was done using previously described spectral components associated with different secondary structures^36,37^. The bands corresponding to the region 1,605–1,620 cm^−1^ are attributed to side chains, 1,620–1,630 cm^−1^ to cross β-sheets, 1,630–1,637 cm^−1^ to parallel β-sheets, 1,638–1,646 cm^−1^ to unordered structures, 1,647–1,662 cm^−1^ to α-helices, 1,662–1,678 cm^−1^ to loops and turns and 1,690–1,697 cm^−1^ to anti-parallel β-sheets. In addition, the band corresponding to the carbonyl group between 1,730–1,760 cm^−1^ was analysed. The bands of the Amide II group at 1,548 and 1,515 cm^−1^ are assigned to α-helices and Tyrosine, respectively. The focus in this analysis was set on the Amide I band. All assigned bands are listed in Table 1. The areas under all peaks were integrated for each spectrum and presented as box plots for N- and C-type in Fig. 4c. In addition, the single values were overlapped to the boxplot in a swarmplot showing the individual distributions of the values. The deconvolution of showed regarding Amide I, that the difference between the two groups of patients is most pronounced for the turns and loops secondary structure with the peak maximum at 1,666 cm^−1^.The analysis further confirmed that protein aggregates in form of fibrils (~ 1,625 cm^−1^) are more prominent in the N-type of cataract. On the other hand, C-type contained more oligomer proteins which have absorbance maximum at 1,693 cm^−1^.

The region between 1,180 and 1,300 cm^−1^ corresponds to the P=O asymmetric band (mostly in nucleic acids of DNA and RNA), shown in Fig. 5. The scores plot shows, that the groups separate strongly along PC1 (Fig. 5c). The loadings of PC1 has strong contributions at 1,220 (minima), 1,245 (maxima), both corresponding to asymmetric P=O band in DNA, and 1,285 cm^−1^ (maxima) connected to Amide III.

Further differences between the two groups of patients were seen in the region between 950 and 1,120 cm^−1^, corresponding to the phosphorylated proteins (970–990 cm^−1^)^39^ and C–O stretch of ribose ring (~ 1,120 cm^−1^)^39^ (Fig. 6). The two groups differ mainly in the PC2 componend (Fig. 6c). The PC2 showed maximum at 1,050 and minimum at 1,080 cm^−1^, corresponding to differences in carbohydrates and glycogen absorbtion ~ 1,050 cm^−1^, and the symmetric stretching P=O bonds (~ 1,080 cm^−1^) in the phosphodiester group between the two groups of patients (Fig. 6b).

### FTIR imaging

Visible light images on Fig. 7 show the curvilinear border of the anterior lens epithelium, as obtained by continuous curvilinear capsulorhexis, on the right upper side for the example of the N cataract type and on the right side for the C cataract type example. A histological distribution of the main biomacromolecular families in the tissues of N and C type of cataract was superimposed on the visible light images by using FTIR imaging. Here matrices of infrared spectra were measured at the regions of interest and presented as the integrals of the major infrared absorption bands of certain biomacromolecules. The FTIR imaging has been performed at the region of the lens epithelium approximately 1.5–2 mm long, located in the central part of the LC (as shown in Fig. 7), which has been removed during the cataract surgery by continuous curvilinear capsulorhexis technique. The matrices with 160 × 800 points and 10 µm spacing between points, allow locating possible changes within individual anterior lens epithelium, from the more central region to its periphery.

Figure 7 shows chemical images for representative samples, C2 and N4 types of cataract. We integrated region of interests i.e. bands where spectroscopic data shoved the most prominent differences (A: the β sheet associated with fibrils aggregates, i.e. (1,620–1,630 cm^−1^), B: ratio of CH_2_ and CH_3_ bands (ν_as_ CH_2_/ν_as_ CH_3_), and C: the ratios of the carbonyl group and asymmetric vibration of both CH_2_ and CH_3_ (ν C=O/ν_as_ CH_2_ + CH_3_)). It is obvious that the large LECs tissues differ from the central point up to periphery, and this inhomogeneity is possible to see in the VIS and the chemical images. However, our spectroscopycal measurements were always set in the central area of each tissue, in order to be comparable between each other. Nevertheless, these results obtained by imaging correspond well with our spectroscopically obtained data. As one can see the N-type contained more proteins aggregates (Fig. 7a), while C-type contained more oxidized lipids (Fig. 7b,c). As for the spectral analysis shown in the previous chapter, N-type of cataract showed also on the FTIR image higher absorption β sheet associated with fibrils aggregates with a maximum at 1625 cm^−1^. On the other hand, in C-type of cataract the increase of lipid oxidative parameter is more pronounced (Fig. 7b,c).

The images indicate that both the higher absorption β sheet associated with fibrils aggregates and the increase of the lipid oxidative parameter were more pronounced in more central part of the anterior lens epithelium.

## Discussion

Here we evaluated and compared the proteins conformation changes, as well as lipids, nucleic acids and carbohydrates bio-macromolecules in human LECs of two different cataract types—N and C cataract. We found that protein aggregation in a form of fibrils is associated with N cataract type LECs, while the oxidative stress and the lipids peroxidation were more pronounced in C cataract type LECs. These significant spectral changes can be assigned to specific biochemical processes occur in two different cataract types LECs, suggesting their functional importance in cataractogenesis. Validating of the cell compounds in human lens epithelia and their comparison between the lens epithelia of N and C cataract patients on the level of single cells was not analysed by SR-FTIR up to know, to the best our knowledge.

Surprisingly, even though high inter-capsule variability was analysed, still the clear statistically relevant differences were found only between N and C cataract types. We compared the different degrees of development of cataracts from the lowest 1st level to the highest 4th level, however, no differences were found in any of the biochemical compounds, which also accentuate that the differences between N and C cataracts are important.

Between the central and peripheral region of the lens epithelia of all studied samples in any of the bio-macromolecular compounds the PCA analysis did not show differences (data not shown). The changes were smaller than those found between different cataract types, which suggest that described changes tend to be homogeniously distributed, implying that the pathological changes of the lens epithelia in C and N cataracts involve changes distributed in the anterior epithelia.

Moreover, the FTIR imaging, i.e. histological distribution of the main bio-macromolecular families in the tissues of C and N types of cataract confirmed the same difference as spectroscopical analysis: N-type of cataract contained higher concentration of β-sheet protein structures, associated with fibrils aggregates while C-type of cataract displayed an increase of lipid oxidative parameters (Fig. 7).

Since the lens epithelia from the control healthy, non cataractous lens is difficult to obtain (the supply of the donor lenses from cadavers was not available), in order to compare with cataract tissues, this study was concentrated on comparison of different cataract types, which is anyhow important for understanding cataractogenesis.

Not much is known about the LECs compounds (proteins, lipids, DNA) changes associated with age-related N and C cataract development. As LECs control energy production, antioxidative mechanisms and biochemical transport for the whole lens, these compound changes are of a high relevance.

Regarding the proteins secondary structures, and possible aggregation types in the cells, the most pronounced difference we have found in lens epithelia of N cataract type was in protein aggregates in the form of fibrils, while in C type only some olygomers structures were observed (Figs. 3 and 4). Numerous human diseases are caused by protein aggregation. However, this is the first study, up to our knowledge, pointing out protein aggregation in LECs in association with N cataract.

There are not many studies where proteins were compared in lens epithelia of N and C cataracts. Kalariya et al. reported an increase in insoluble proteins only in the C cataractous epithelium, while in the N cataractous epithelium the changes were negligible^44^. However, the FTIR analysis describes total protein changes, without pointing out to one specific protein, which could make aggregates. The post-translational protein crosslinking is catalyzed by the enzyme Transglutaminase2 (TG2) and in human LECs cell line (HLE-B3) oxidative stress or UV radiation were shown to induce an aberrant in situ TG2 activation^45^. The up-regulation and activation of TG2 have been reported in cataractogenesis^46^ suggesting the importance of LECs proteins structural changes in cataractogenesis.

LECs and differentiated fiber cells represent distinct compartments in the ocular lens. We have previously provided detailed evidence about the structural organization of the LECs^47^. LECs have the nucleus and all the organelles preserved. The endoplasmic reticulum (ER) is the site of protein synthesis and protein folding into proper structures^48,49^. Only properly folded proteins are transferred to the Golgi complex for further modification^50^.

Cataracts can be caused by many stressors, including ER stressors^51^ , which in lens give rise to the accumulation of unfolded protein aggregates^52–55^. Many different intracellular stress pathways converge at the unfolded protein response^55,56^, which is induced by unfolded protein aggregates in the ER, after exposure to environmental changes that create ER stress^57^. We can speculate that there might be an error in its regulation in N cataract LECs.

In connection with the proteins aggregates in LECs, αA and αB crystallins, which are known to be a members of the small heat shock protein family of molecular chaperones, can prevent non-specific aggregation of denaturing proteins^58^. α crystallins can protect LECs from stress-induced cell death and from environmental stress^59–61^ and thus may delay the onset of age-related cataract^62^. In the lens epithelium both mRNA and soluble protein expressions of αA and αB crystallins were reduced in age-related cataract group^63^. β and γ crystallins are normally abundant in adult mammalian LECs and there is a complex regulation of the accumulation of crystallin mRNAs in LECs after stress and at different ages^64^. Most proteins, including lens crystallins, have β-sheets as part of their molecular conformation^65^. Not much is known about crystallins in lens epithelia of N and C cataract. We hipotised that in the lens epithelia of N cataract patients, α crystallins protective function in preventing non-specific aggregation of denaturing proteins could be decreased or abolished. Siamwiza and coworkers showed Tyr residues as sensitive for proteins’ secondary structure changes. This is associated with the formation of strong hydrogen bonds with acceptor by Tyr rather than with H_2_O under the influence of appearing aggregation^66,67^. This is in agreement with our study.

An important difference that we have found between lens epithelia of N and C cataract types was in the lipid peroxidation and oxidative stress effects on lipid structures. However, LECs are recognised and studied as the targets of oxidative stress acting on lipids. It was shown that oxidative stress leads to changes in membrane composition in human LECs^68^. Mitochondrial induced oxidative stress was suggested to enhance lipid peroxidation and cellular membrane damage in LECs^17,69,70^. Besides, lipids composition of lens epithelial membranes was different than fiber cell membranes and it was found to contain more glycerolipids and less sphingolipids by Raman spectroscopy^71^.

Considering that the LECs are metabolically the most active part of the lens with the LECs having all the organells and considering that lipids are key components of the plasma membrane and other cellular compartments, including the nuclear membrane, ER, Golgi apparatus and trafficking vesicles and that the structure of lipids can affect membrane properties and functions such as membrane permeability and kinetics of enzymatic processes^72^, importance of studying lipids in LECs is obvious. Lipid imbalances are connected with many cases of pathologies^73^. The peroxidation of lipids in biological membranes has been implicated in the onset and development of most degenerative diseases^74^.

The fact that the oxidative stress and the lipids peroxidation are more pronounced in C cataract type LECs is in a line with that the C cataracts are associated with mutations and lens epithelial changes, particularly following UV and IR exposure^16^. This is in agreement with the study showing that the hydrocarbon chain disorder was estimated to be 72 and 58% for the C and the N lipids^75^.

Regarding DNA and nucleic acids, we have found the biggest differences between lens epithelia of N and C cataract types regarding the asymmetric and symmetric phosphate bands, mostly corresponding to DNA and phosphorylated proteins by using the PCA analysis (Figs. 5 and 6). However, it is difficult to estimate if the DNA in the LECs epithelial was damaged. Zhang and colleague did not show the difference in DNA damage between the N and C lens epithelia^76^. From the other side, Sorte et al*.* reported that the DNA damage in LECs was found maximally in the C type of cataracts^77^. Still, more research groups showed that DNA damage in LECs is connected with cataract. In age-related cataract compared to control group LECs DNA damage was shown to increase^76^. DNA single strand breaks were found in human LECs from cataract patients^78^. The fact that the lens epithelium is the lens region having the nucleus and DNA reflects the importance of DNA being non-damaged for the health of the lens. We can speculate that both mechanisms involved in N and C cataract formation include DNA damage.

## Conclusion

Cataract is a result of the functional impairment of two constitutive types of lens cells, LECs and fiber cells. Here we present an interconnection between damage of the lens epithelium cells and the developmental types of lens opacities, and the correlation with changes on the level of the essential bio-macromolecules. Our results obtained by FTIR increase the pale knowledge about the total proteins, lipids and nucleic acids changes in single cells in lens epithelium in connection with N and C cataract. We have clearly shown that protein aggregation in the form of fibrils was prominent in LECs of N cataracts, while oxidative stress and the lipids peroxidation were more pronounced in LECs of C cataracts. Further research on the lens epithelia in connection with N and C cataract development is necessary in order to understand the cataractogenesis and to search for right prevention or potential treatment of these diseases with different macromolecular changes.

## Data Availability

The datasets generated during and/or analysed during the current study are available from the corresponding author on reasonable request.
